# Exploring the Number of Web-Based Behavioral Health Coaching Sessions Associated With Symptom Improvement in Youth: Observational Retrospective Analysis

**DOI:** 10.2196/52804

**Published:** 2023-12-18

**Authors:** Darian Lawrence-Sidebottom, Landry Goodgame Huffman, Aislinn Beam, Rachael Guerra, Amit Parikh, Monika Roots, Jennifer Huberty

**Affiliations:** 1 Bend Health, Inc Beaverton, OR United States; 2 FitMinded, Inc LLC Phoenix, AZ United States

**Keywords:** adolescents, anxiety, children, depression, digital mental health intervention, reliable change

## Abstract

**Background:**

Rates of anxiety and depression have been increasing among children and adolescents for the past decade; however, many young people do not receive adequate mental health care. Digital mental health interventions (DMHIs) that include web-based behavioral health coaching are widely accessible and can confer significant improvements in youth anxiety and depressive symptoms. However, more research is necessary to determine the number of web-based coaching sessions that confer clinically significant improvements in anxiety and depressive symptoms in youth.

**Objective:**

This study uses data from a pediatric DMHI to explore the number of web-based coaching sessions required to confer symptom improvements among children and adolescents with moderate or moderately severe symptoms of anxiety and depression.

**Methods:**

We used retrospective data from a pediatric DMHI that offered web-based behavioral health coaching in tandem with self-guided access to asynchronous chat with practitioners, digital mental health resources, and web-based mental health symptom assessments. Children and adolescents who engaged in 3 or more sessions of exclusive behavioral health coaching for moderate to moderately severe symptoms of anxiety (n=66) and depression (n=59) were included in the analyses. Analyses explored whether participants showed reliable change (a decrease in symptom scores that exceeds a clinically established threshold) and stable reliable change (at least 2 successive assessments of reliable change). Kaplan-Meier survival analyses were performed to determine the median number of coaching sessions when the first reliable change and stable reliable change occurred for anxiety and depressive symptoms.

**Results:**

Reliable change in anxiety symptoms was observed after a median of 2 (95% CI 2-3) sessions, and stable reliable change in anxiety symptoms was observed after a median of 6 (95% CI 5-8) sessions. A reliable change in depressive symptoms was observed after a median of 2 (95% CI 1-3) sessions, and a stable reliable change in depressive symptoms was observed after a median of 6 (95% CI 5-7) sessions. Children improved 1-2 sessions earlier than adolescents.

**Conclusions:**

Findings from this study will inform caregivers and youth seeking mental health care by characterizing the typical time frame in which current participants show improvements in symptoms. Moreover, by suggesting that meaningful symptom improvement can occur within a relatively short time frame, these results bolster the growing body of research that indicates web-based behavioral health coaching is an effective form of mental health care for young people.

## Introduction

Between 2016 and 2019, rates of anxiety and depression in youth increased dramatically, with 5.8 million children and adolescents diagnosed with anxiety and 2.7 million with depression [1]. However, 1 in 5 young people do not receive adequate mental health treatment [1], highlighting a crucial need for greater accessibility in pediatric mental health services. Digital mental health interventions (DMHIs) have emerged as scalable and accessible alternatives to traditional forms of mental health care [2,3]. DMHIs, which encompass a range of mental health services administered through electronic modalities such as digital video chat, smartphone apps, and chat rooms [4], can confer comparable therapeutic benefits while also presenting fewer barriers to care [5]. Behavioral health coaching, which provides nonclinical and goal-focused mental health care to those with mild to moderate symptoms [6], is often more accessible and affordable than traditional therapy [7,8]. Moreover, DMHIs that use web-based behavioral health coaching can confer significant decreases in adult anxiety and depressive symptoms, as well as greater adherence, when compared with purely self-guided digital interventions [9-13]. Preliminary evidence also suggests that web-based behavioral health coaching is linked to decreases in anxiety and depressive symptoms for children and adolescents participating in a pediatric DMHI [14].

Although it is well known that greater engagement (eg, more sessions and longer participation) in DMHIs that include web-based coaching is linked to increased therapeutic benefits [14-17], more research is necessary to identify the number of web-based coaching sessions that confer clinically significant improvements in anxiety and depressive symptoms in youth. Understanding these nuances in care will not only enable comparisons between DMHIs and traditional in-person interventions but also help caregivers gauge the time and commitment necessary to see meaningful changes in their children [18]. Therefore, this study aims to provide a preliminary exploration of the number of web-based coaching sessions required to confer symptom improvements among children and adolescents with moderate or moderately severe symptoms of anxiety and depression.

## Methods

### Participants

Bend Health Inc members (aged between 6 and 17 years) participating in behavioral health coaching only (no therapy) for at least 3 months between January and August 2023 were eligible for inclusion in the study (N=392).

### Treatment

As described previously [14,17], Bend Health Inc is a pediatric DMHI in which child and adolescent members engage in the following services: digital behavioral video conferencing (synchronous) sessions with mental health providers, a web-based learning resource center, web-based asynchronous messaging with mental health providers (caregiver login), and mental health symptom assessments. Most members are referred by their primary care provider, or they access Bend through insurance, employer benefits, or direct-to-consumer pathways. Each member is assigned a behavioral care manager, who is responsible for overseeing a member’s progress and integrating their care between mental health and medical providers. While members may attend coaching or therapy sessions, only members in exclusive coaching were included in this study. Bend coaches are certified behavioral coaches or masters-level mental health providers trained in standard coaching techniques. During the 30-minute coaching sessions, coaches guide members and their families through structured care programs, which are intended to provide evidence-based behavior-change tools to target a particular mental health symptom. Coaches use tools based on cognitive behavioral therapy, parent management training, behavioral activation, mindfulness-based cognitive therapy, mindfulness-based stress reduction, and motivational interviewing. Members also have access to the content of their care program in a learning resource center, where they can practice and refresh their skills in an asynchronous format. Furthermore, caregivers may message their child’s mental health providers through a secure web-based portal. Some members also receive care from a psychiatric provider, depending on insurance benefits, primary care provider referral, and mental health symptom acuity.

### Measures

At enrollment with Bend Health Inc, caregivers report their child or adolescent’s date of birth, sex at birth, gender, and race or ethnicity, as described elsewhere [14]. Mental health screening questions are also completed by caregivers of children and adolescents at enrollment. These questions are from the *Diagnostic and Statistical Manual of Mental Disorders, Fifth Edition* (DSM-5), cross-cutting measures for Level 1 caregiver-report (children) and self-report (adolescent) [19], which ask about the frequency of symptoms in the past 2 weeks. Then, full validated assessments are completed to thoroughly assess symptoms flagged by the screening questions using established scoring criteria.

For children, the full caregiver-reported assessments for anxiety and depressive symptoms are the PROMIS (Patient Reported Outcomes Measurement Information System) measures [20,21], which are 10-items and 11-items, respectively. For adolescents, the full self-reported anxiety symptom assessment is the Generalized Anxiety Disorder 7-item (GAD-7) measure [22], and the full depressive symptom assessment is the Patient Health Questionnaire 9-item adolescent (PHQ-9A) version [23]. The question about suicide was removed from the PHQ-9A in this study, so it was 8 items in length. Assessments (screeners and full assessments) are repeated approximately monthly during care. Further details on the demographic information collected at enrollment, as well as mental health screener questions and scoring criteria, can be found in Multimedia Appendix 1.

### Statistical Analysis

To ensure that study participants had similar care characteristics, the inclusion criteria listed in Textbox 1 were used:

**Inclusion criteria for the study participants.**

**Inclusion criteria**

- 2 completed assessments (baseline and during coaching; n=24 excluded)
- 1.5-2.5 sessions per month (n=101 excluded)
- 1-3 weeks between-sessions (n=77 excluded)
- 3 or more sessions attended (n=37 excluded)

Scores from each assessment were aggregated, and then, using standard methods, PROMIS assessment scores were converted to T-scores [10,11], and PHQ-9A scores were adjusted to account for the removed item [23]. Aggregate GAD-7 scores were not converted. Only the first assessment between sessions was retained, and baseline symptom severity was assessed before coaching using established criteria [20-23]. Final analyses were performed using data from members with anxiety or depressive symptoms of moderate or moderately severe severity at baseline. Member characteristics and care statistics (duration in coaching, sessions per month, and between-session duration) were reported for the anxiety and depressive symptoms groups.

The reliable change criterion (RCC) was calculated for each validated assessment using published values for measure validity and data from all Bend Health Inc members at enrollment in 2023 for measure variance (Table S1 in Multimedia Appendix 1 contains calculated values) [24]. Then, scores from each assessment during coaching were compared against the corresponding RCC. A participant showed reliable change if (1) their scores decreased from baseline by a value greater than the RCC or (2) they screened out of the full assessment. A participant had stable reliable change when they completed 2 successive assessments with reliable change. Kaplan-Meier survival analyses were performed to determine the median number of coaching sessions when the first reliable change and stable reliable change occurred for anxiety and depressive symptoms [25]. For members with no change, the total number of coaching sessions attended was used in the survival analyses. Follow-up group-wise survival analyses were repeated on a children-only subsample (aged between 6 and 12 years) and an adolescent-only subsample (aged between 13 and 17 years) to assess for differences in age and assessment type.

### Ethical Considerations

All study protocols were reviewed and approved by the Biomedical Research Alliance of New York (Study 23-12-034-1374). Informed consent was obtained at enrollment for participation with Bend Health Inc services after an overview of the services, data use, and privacy policy. Caregivers consented on behalf of child members (aged between 1 and 12 years), and adolescent members (aged between 13 and 17 years) consented on their own behalf. All human participant data were deidentified before analysis. Study participants received no compensation for their inclusion in the study.

## Results

### Member Characteristics and Participation in Care

There were 66 members in the anxiety symptoms group and 59 members in the depressive symptoms group. Comprehensive member demographics are reported in Table 1. Members completed between 2 and 7 assessments (Table 2; the anxiety symptoms group completed a median of 4 [IQR 3-5] assessments, and the depressive symptoms group completed a median of 4 [IQR 3-4.5] assessments). Members participated in coaching for 3.00-7.23 months, attending 3-17 sessions (Table 3). Members in the anxiety symptoms group were in coaching for 4.32 (SD 1.07) months, and they attended a median of 7 (IQR 5.25-10.0) coaching sessions. Members in the depressive symptoms group were in coaching for 4.34 (SD 1.09) months, and they attended a median of 7 (IQR 6-9) coaching sessions. The anxiety symptoms group had 1.97 (SD 0.30) sessions per month (2.19, SD 0.37 weeks between sessions). The depressive symptoms group had 1.99 (SD 0.29) sessions per month (2.19, SD 0.37 weeks between sessions).

**Table 1:** Demographic characteristics of members in the analyses for anxiety symptoms and elevated depressive symptoms.

Demographic			Anxiety symptoms (n=66)		Depressive symptoms (n=59)
Age (years), mean (SD)			11.0 (3.2)		12.4 (3.1)
Child, n (%)			43 (65)		26 (44)
Adolescent, n (%)			23 (35)		33 (56)
**Sex, n (%)**					
	Female	34 (52)		33 (56)	
	Male	31 (47)		25 (42)	
	Other	1 (2)		1 (2)	
**Gender conformity, n (%)**					
	Conforming	63 (96)		57 (97)	
	Nonconforming	3 (4)		2 (3)	
**Ethnicity, n (%)**					
	Asian	4 (6)		2 (3)	
	Black or African American	1 (2)		0 (0)	
	Hispanic or Latino	2 (3)		4 (7)	
	White	30 (46)		33 (56)	
	Other or multiracial	29 (44)		20 (34)	

**Table 2:** Members that completed assessments 1-6 by symptom group.

Assessment	Anxiety symptoms (n=66), n (%)	Depressive symptoms (n=59), n (%)
1	66 (100)	59 (100)
2	66 (100)	59 (100)
3	61 (92)	56 (95)
4	41 (62)	37 (63)
5	21 (32)	15 (25)
6	6 (9)	2 (3)

**Table 3:** Members that attended each coaching session by symptom group.

Coaching session	Anxiety symptoms (n=66), n (%)	Depressive symptoms (n=59), n (%)
3	66 (100)	59 (100)
4	65 (98)	55 (93)
5	56 (85)	52 (88)
6	49 (74)	46 (78)
7	38 (58)	33 (56)
8	27 (41)	25 (42)
9	20 (30)	19 (32)
10	19 (29)	14 (24)
11	12 (18)	7 (12)
12	8 (12)	3 (5)
13	4 (6)	2 (3)
14	2 (3)	1 (2)
15	1 (2)	0 (0)

### Anxiety Symptoms: Time to Improvement

**Figure 1:**
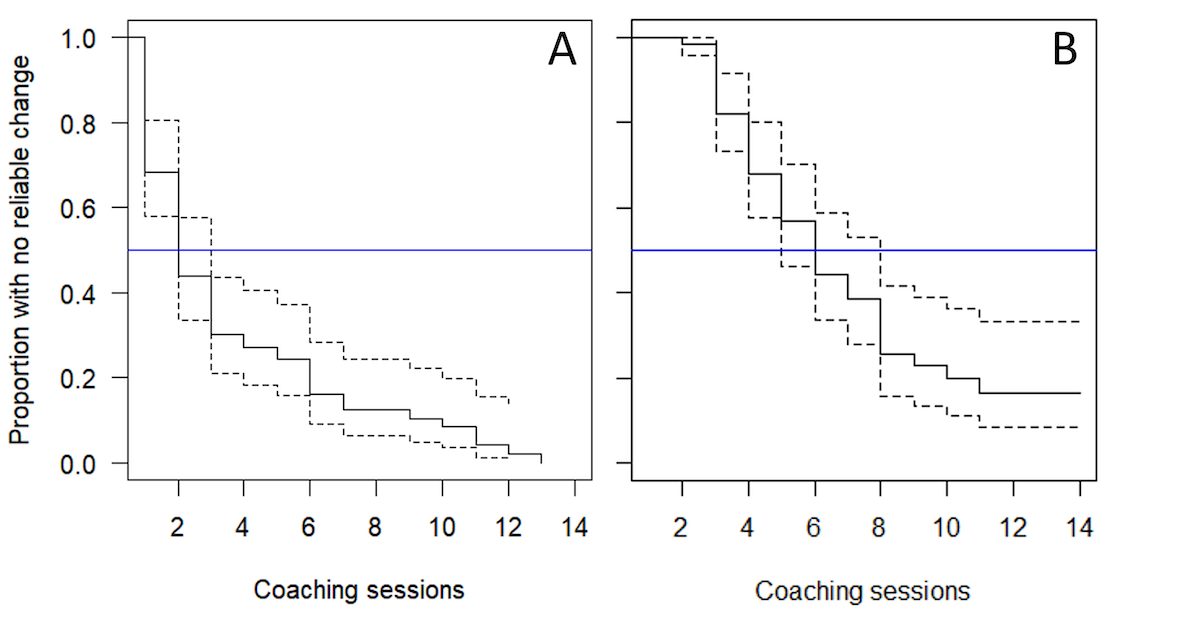
Survival plots for anxiety symptoms over coaching sessions. The solid black line indicates proportion with no reliable change (survival). The dotted lines indicate the CI. The blue line on the y-axis indicates a proportion of 0.5. A: Reliable change. B: Stable reliable change.

For anxiety symptoms over coaching with the DMHI, a total of 96% (63/66) of members had reliable improvements, and 71% (47/66) of members exhibited stable reliable improvements (Table S2 in Multimedia Appendix 1). Reliable change in anxiety symptoms was observed after a median of 2 (95% CI 2-3) sessions, and stable reliable change in anxiety symptoms was observed after a median of 6 (95% CI 5-8) sessions (Figure 1). For children, reliable change was observed after a median of 2 (95% CI 2-3) sessions, and stable reliable change was observed after a median of 6 (95% CI 4-8) sessions. For adolescents, reliable change was observed after a median of 3 (95% CI 2-7) sessions, and stable reliable change was observed after a median of 6 (95% CI 5-not available) sessions.

### Depressive Symptoms: Time to Improvement

**Figure 2:**
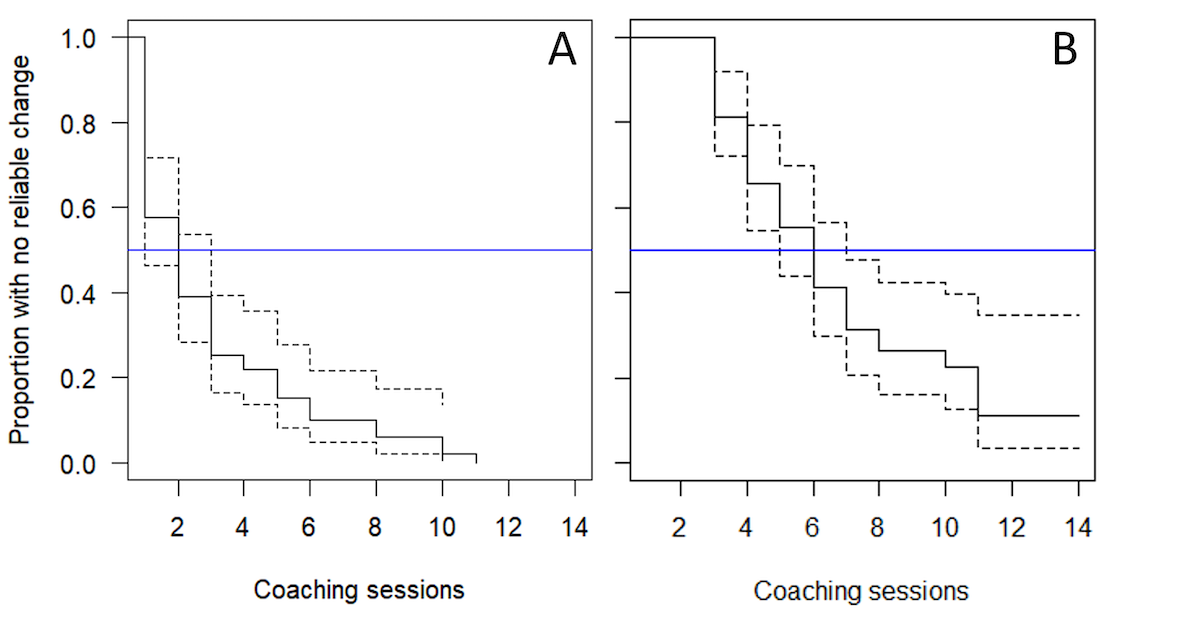
Survival plots for depressive symptoms over coaching sessions. The solid black line indicates proportion with no reliable change (survival). The dotted lines indicate the CI. The blue line on the y-axis indicates a proportion of 0.5. A: Reliable change. B: Stable reliable change.

For depressive symptoms over coaching with the DMHI, a total of 98% (58/59) of members had reliable improvements, and 71% (42/59) of members exhibited stable reliable improvements (Table S3 in Multimedia Appendix 1). Reliable change in depressive symptoms was observed after a median of 2 (95% CI 1-3) sessions, and stable reliable change in depressive symptoms was observed after a median of 6 (95% CI 5-7) sessions (Figure 2). For children, reliable change was observed after a median of 1.5 (95% CI 1-2) sessions, and stable reliable change was observed after a median of 5 (95% CI 4-11) sessions. For adolescents, reliable change was observed after a median of 3 (95% CI 1-4) sessions, and stable reliable change was observed after a median of 6 (95% CI 5-10) sessions.

## Discussion

### Principal Findings

The purpose of this study was to provide a preliminary exploration of the number of web-based coaching sessions associated with clinically significant reductions in symptoms among children and adolescents with moderate to moderately severe symptoms of anxiety and depression. For both anxiety and depressive symptoms, most members showed reliable symptom improvements after 2 coaching sessions and stable improvements after 6 coaching sessions. Children improved 1-2 sessions earlier than adolescents. This is the first study to suggest that reliable and stable changes in anxiety and depressive symptoms can be observed for most participants in a pediatric DMHI after just six 30-minute web-based coaching sessions.

Although preliminary, these findings provide valuable information to those seeking pediatric mental health treatment for anxiety and depressive symptoms. There are many barriers to accessing mental health care, with affordability being one of them [26]. If caregivers are able to anticipate the number of sessions that will likely lead to improvements in their children’s symptoms, they may be more willing and able to make the financial commitment to their children’s treatment. Additionally, when caregivers and youth have a better understanding of the approximate treatment length, they may be more likely to continue attending their coaching appointments when they encounter factors commonly associated with early dropout from mental health treatment (eg, lack of stability, lack of motivation, symptom severity, or parental stress) [27-29]. Indeed, several studies of traditional therapeutic modalities have found that clients typically attend the number of sessions they expect to attend, suggesting that a realistic expectation for participation will maximize families’ engagement in their children’s mental health care [29-31].

### Comparison With Previous Work

Adolescents had improvements in symptoms about 1-2 more sessions later than children. While the participants in this study all had moderate or moderately severe symptom severity at baseline, other studies comparing child and adolescent responses to clinical behavioral interventions have found that adolescents typically exhibit more severe symptoms and a less favorable treatment response than children [32,33]. Older youth with anxiety or depressive symptoms may also experience increased distress as their symptoms interact with socioemotional and environmental changes characteristic of adolescence, such as increased autonomy and greater stress at school. Alternatively, this marginal age-based difference may reflect biases in caregiver versus self-report or differences in the sensitivities of the measures used to assess symptoms in children and adolescents. Although further study is necessary to replicate our results, these preliminary findings suggest the advantage of tailoring pediatric DMHI programs to age.

### Limitations

Our findings are limited by several factors. Given that the reliable change criteria used for the measures in this study were relatively small, further study is necessary to confirm whether 6 coaching sessions consistently confer substantive improvements in mental health symptoms. However, our results are consistent with a systematic review, which found that low-intensity in-person psychotherapy yielded optimal results between 4 and 6 sessions [34]. This study also did not include a nonactive control group; therefore, we could not assess whether coaching alone produced improvements in mental health symptoms. Future studies would be bolstered by the inclusion of a control group to address this limitation. Moreover, we were unable to quantify changes in symptoms for members who screened out of taking the full assessments at later time points. We addressed this potential bias by also assessing stable reliable change as a measure of symptom improvement, thus ensuring our results were not driven by those with screened-out assessments. Finally, we assessed symptom severity approximately every 30 days, rather than after every coaching session. Given that members completed approximately 2 coaching sessions per month, symptom improvements after the coaching sessions between assessments were not reflected in the present results. Therefore, future studies would benefit from improved measurement and timing of mental health symptom assessments.

### Conclusions

This study provides compelling preliminary evidence that participation in 2-6 web-based coaching sessions is associated with reliable improvements among youth with moderate or moderately severe anxiety and depressive symptoms. Findings from this study inform caregivers and youth seeking mental health care by characterizing the typical time frame in which current participants show improvements in symptoms. Moreover, by suggesting that meaningful symptom improvement can occur within a relatively short time frame, these results bolster the growing body of research that indicates web-based behavioral health coaching is an effective form of mental health care for young people.

## Appendix

Multimedia Appendix 1 Supplementary material and tables.

## Abbreviations

DMHI: digital mental health intervention
DSM-5: Diagnostic and Statistical Manual of Mental Disorders, Fifth Edition
GAD-7: Generalized Anxiety Disorder 7-item
PHQ-9A: Patient Health Questionnaire 9-item adolescent
PROMIS: Patient Reported Outcomes Measurement Information System
RCC: reliable change criterion

## Data Availability

The data sets analyzed during this study are not publicly available, as this would violate Bend Health Inc’s privacy policy, but they are available from the corresponding author on reasonable request.
