# Supplementary material for: Exploring the Number of Web-Based Behavioral Health Coaching Sessions Associated With Symptom Improvement in Youth: Observational Retrospective Analysis
Source: JMIR Form Res. 2023 Dec 18;7:e52804. doi: 10.2196/52804 (PMC10758935; doi:10.2196/52804)
Supplement: Multimedia Appendix 1 [file formative_v7i1e52804_app1.docx]

Multimedia Appendix 1

**Methods**

Caregivers select their member’s demographic information from drop-down options at enrollment. For sex (at birth), the options are: Male, Female, and Other. For gender, the options are: Male, Female, Transgender, Non-Binary, and Other. In determining gender conformity, members with the same sex and gender are reported here as gender conforming and members with a different sex and gender are reported here as gender non-conforming. For race/ethnicity, the drop-down options from January 1st 2023 to May 25th 2023 were: American Indian or Alaska Native, Asian, Black or African American, Hispanic or Latino, Native Hawaiian or Other Pacific Islander, White, and Other. Beginning May 26th 2023, the race/ethnicity options are: White, Black or African American, American Indian or Alaska Native, Chinese, Vietnamese, Native Hawaiian, Filipino, Korean, Japanese, Chamorro, Other Asian, Other Pacific Islander, Some other race or multi-racial, Mexican or Mexican American or Chicano, Puerto Rican, Cuban, and Another Hispanic, Latino, or Spanish origin. Also beginning May 26th 2023, caregivers are able to select more than one race/ethnicity option (multi-select). Given the change in the race/ethnicity options part-way through the study, as well as the low representation for many minority groups, the following categories were used here to describe member race/ethnicity (response options included in parentheses): White (White), Hispanic/Latino (Hispanic or Latino, Other Hispanic or Latino, Mexican or Mexican American or Chicano, Puerto Rican, and Cuban), Asian (Chinese, Vietnamese, Filipino, Korean, Japanese, Chamorro, Other Pacific Islander, and Other Asian), Black or African American (Black or African American), and Other (Other or Multiracial, and multi-select responses).

To screen for child anxiety symptoms, caregivers respond to the following three questions about their child’s behavior over the last two weeks: 1) “said they felt nervous, anxious, or scared,” 2) “not been able to stop worrying,” and 3) “said they couldn't do things they wanted to or should have done, because it made them feel nervous.” To screen for child depressive symptoms, caregivers respond to the following two questions: 1) “had less fun doing things than she used to”, and 2) “seemed sad or depressed for several hours.” For the anxiety and depressive screener questions, responses were made using a five-item Likert-type scale (Not at all [0] to Nearly every day [4]). For child anxiety and depressive symptoms, caregivers are prompted to take the full corresponding symptom assessment if they respond to any question with a two or greater (several days or more frequent). To screen for adolescent anxiety symptoms, adolescents respond to the following two questions about their behavior and feelings over the last two weeks: 1) “feeling nervous, anxious, or on edge,” 2) “not being able to stop or control worrying.” To screen for adolescent depressive symptoms, adolescents respond to the following two screening questions about their behavior and feelings over the last two weeks: 1) “had less fun doing things than you used to”, 2) “felt sad or depressed for several hours.” All responses to the adolescent screening questions are on a four-item Likert-type scale (Not at all [0] to Nearly every day [3]). For both anxiety and depressive symptoms, if the sum of the two screener questions is two or more, the adolescent is prompted to complete the full corresponding assessment.

**Tables**

**Table S1.** Values used in the calculation of the reliable change criterion (RCC) for each symptom assessment.

| **Validated assessment** | **SD (Bend members used in calculation)** | **Chronbach's alpha (Reliability)** | **Reliable change index (RCI)** | **Reliable change criterion (RCC)** |
| --- | --- | --- | --- | --- |
| PROMIS -  Child anxiety | 8.73 (n = 768) | 0.93 | 2.31 | 4.53 |
| GAD-7 -  Adolescent anxiety | 4.70 (n = 608) | 0.94 | 1.94 | 3.80 |
| PROMIS -  Child depressive | 9.43 (n = 453) | 0.83 | 2.31 | 4.53 |
| PHQ-9A -  Adolescent depressive | 5.47 (n = 499) | 0.86 | 2.05 | 4.01 |

**Table S2.** Results from the Kaplan-Meier survival analyses of reliable change (left side of table) and reliable and stable change (right side of table) in anxiety symptoms.

|  | **Reliable change** | | | **Stable reliable change** | | |
| --- | --- | --- | --- | --- | --- | --- |
| **Coaching sessions** | **Risk:** Members with no change  % (n) | **Event:** Members with change  % (n) | **Survival:** Cumulative members no change  % | **Risk:** Members with no change  % (n) | **Event:** Members with change  % (n) | **Survival:** Cumulative members no change  % |
| 1 | 100.0%  (n = 66) | 31.8%  (n = 21) | 68.1% | 100.0%  (n = 66) | 0.0%  (n = 0) | 100.0% |
| 2 | 68.2%  (n = 45) | 24.2%  (n = 16) | 43.9% | 100.0%  (n = 66) | 1.5%  (n = 1) | 98.5% |
| 3 | 43.9%  (n = 29) | 13.6%  (n = 9) | 30.0% | 98.5%  (n = 65) | 16.7%  (n = 11) | 81.8% |
| 4 | 30.3%  (n = 20) | 3.0%  (n = 2) | 27.3% | 80.3%  (n = 53) | 9.1%  (n = 6) | 67.9% |
| 5 | 27.3%  (n = 18) | 3.0%  (n = 2) | 24.2% | 65.2%  (n = 43) | 10.6%  (n = 7) | 56.9% |
| 6 | 22.7%  (n = 15) | 7.6%  (n = 5) | 16.2% | 48.5%  (n = 32) | 10.6%  (n = 7) | 44.4% |
| 7 | 13.6%  (n = 9) | 3.0%  (n = 2) | 12.6% | 33.3%  (n = 22) | 4.5%  (n = 3) | 38.4% |
| 8 | 9.1%  (n = 6) | 0.0%  (n = 0) | 12.6% | 22.7%  (n = 15) | 7.6%  (n = 5) | 25.6% |
| 9 | 9.1%  (n = 6) | 1.5%  (n = 1) | 10.5% | 13.6%  (n = 9) | 1.5%  (n = 1) | 22.7% |
| 10 | 7.6%  (n = 5) | 1.5%  (n = 1) | 8.4% | 12.1%  (n = 8) | 1.5%  (n = 1) | 19.9% |
| 11 | 6.1%  (n = 4) | 3.0%  (n = 2) | 4.2% | 9.1%  (n = 6) | 1.5%  (n = 1) | 16.6% |
| 12 | 3.0%  (n = 2) | 1.5%  (n = 1) | 2.1% | 9.1%  (n = 6) | 0.0%  (n = 0) | 16.6% |
| 13 | 1.5%  (n = 1) | 1.5%  (n = 1) | 0.0% | 9.1%  (n = 6) | 0.0%  (n = 0) | 16.6% |

**Table S3.** Results from the Kaplan-Meier survival analyses of reliable change (left side of table) and reliable and stable change (right side of table) in depressive symptoms.

|  | **Reliable change** | | | **Stable reliable change** | | |
| --- | --- | --- | --- | --- | --- | --- |
| **Coaching sessions** | **Risk:** Members with no change  % (n) | **Event:** Members with change  % (n) | **Survival:** Cumulative members no change  % | **Risk:** Members with no change  % (n) | **Event:** Members with change  % (n) | **Survival:** Cumulative members no change  % |
| 1 | 100.0%  (n = 59) | 42.4%  (n = 25) | 57.6% | 100.0%  (n = 59) | 0.0%  (n = 0) | 100.0% |
| 2 | 57.6%  (n = 34) | 18.6%  (n = 11) | 39.0% | 100.0%  (n = 59) | 0.0%  (n = 0) | 100.0% |
| 3 | 39.0%  (n = 23) | 13.6%  (n = 8) | 25.4% | 100.0%  (n = 59) | 18.6%  (n = 11) | 81.4% |
| 4 | 25.4%  (n = 15) | 3.4%  (n = 2) | 22.0% | 79.7%  (n = 47) | 15.3%  (n = 9) | 65.8% |
| 5 | 22.0%  (n = 13) | 6.8%  (n = 4) | 15.3% | 64.4%  (n = 38) | 10.2%  (n = 6) | 55.4% |
| 6 | 15.3%  (n = 9) | 5.1%  (n = 3) | 10.2% | 45.8%  (n = 27) | 11.9%  (n = 7) | 41.0% |
| 7 | 8.5%  (n = 5) | 0.0%  (n = 0) | 10.2% | 28.8%  (n = 17) | 6.8%  (n = 4) | 31.4% |
| 8 | 8.5%  (n = 5) | 3.4%  (n = 2) | 6.1% | 20.3%  (n = 12) | 3.4%  (n = 2) | 26.1% |
| 9 | 5.1%  (n = 3) | 0.0%  (n = 0) | 6.1% | 11.9%  (n = 7) | 0.0%  (n = 0) | 26.1% |
| 10 | 5.1%  (n = 3) | 3.4%  (n = 2) | 2.0% | 11.9%  (n = 7) | 1.7%  (n = 1) | 22.4% |
| 11 | 1.7%  (n = 1) | 1.7%  (n = 1) | 0.0% | 6.8%  (n = 4) | 3.4%  (n = 2) | 11.2% |
